# Quantitative Analysis of Contrast-Enhanced Ultrasound That Can Be Used to Evaluate Angiogenesis during Patellar Tendon Healing in Rats

**DOI:** 10.1155/2022/6867743

**Published:** 2022-10-13

**Authors:** Haojun Zhao, Dandan Sheng, Yehua Cai, Yanlin Tang, Mei Li, Jun Chen, Shiyi Chen, Ping Xu

**Affiliations:** ^1^Department of Ultrasound, Huashan Hospital, Fudan University, Shanghai, China; ^2^Department of Ultrasound, Jing'an District Central Hospital, Fudan University, Shanghai, China; ^3^Department of Sports Medicine, Huashan Hospital, Fudan University, Shanghai, China; ^4^Department of Preventive Medicine and Public Health Laboratory Science, School of Medicine, Jiangsu University, Zhenjiang, China; ^5^State Key Laboratory of Molecular Engineering of Polymers, Department of Macromolecular Science, Fudan University, Shanghai, China

## Abstract

**Objective:**

To investigate the efficacy of contrast-enhanced ultrasound (CEUS) in quantitatively evaluating angiogenesis during patellar tendon healing in rats.

**Methods:**

A total of 40 Sprague–Dawley rats were used in this study. The patellar tendons of 30 rats (60 limbs) that underwent incision and suture were treated as the operation group and monitored after 7, 14, and 28 days. The normal patellar tendons of 10 rats (20 limbs) were treated as the control group and monitored on day 0. The ultrasound examination was used to evaluate the structure and blood perfusion of the patellar tendon. Immunohistochemistry was used to assess angiogenesis, and the biomechanical test was used to verify functional recovery of the patellar tendon.

**Results:**

The tendons in the operation group were significantly thickened compared with those in the control group (*p* < 0.01). The peak intensity (PI) in CEUS of the tendons showed a clear difference at each time point after the surgery (*p* < 0.01). PI increased in the operation group with a maximum on day 7, and then gradually decreased until day 28 when PI was close to the basic intensity (BI) in the control group (*p* > 0.05). It was consistent with the change of the CD31-positive staining areas representing angiogenesis of the injured patellar tendons. The PI was positively correlated with the CD31-positive staining area fraction (*R* = 0.849, *p* < 0.001). The failure load and tensile strength of the repaired patellar tendons in the operation group increased over time. The PI showed negative correlations with the failure load (*R* = −0.787, *p* < 0.001) and tensile strength (*R* = −0.714, *p* < 0.001).

**Conclusion:**

The PI in CEUS could quantitatively reflect the time-dependent change in the blood supply of the healing site, and the PI correlated with histologic and biomechanical properties of the healing tendon. Quantitative analysis of contrast-enhanced ultrasound could be a useful method to evaluate angiogenesis in healing tendons.

## 1. Introduction

Tendon and ligament injuries accounted for 30% of all musculoskeletal consultations, with 4 million new incidences worldwide each year, thus imposing a significant burden on society and the economy [1]. Due to hypovascularity of the tendon tissue, the repair process is slow and inefficient after tendon injury [2]. Therefore, the recovery of blood supply is essential during the repair period of injured tendons. On the other hand, it was reported that prolonged hypervascularization following tendon injury might not be beneficial for healing [3, 4]. Hence, it is believed that clinically in vivo detection of angiogenesis is crucial for evaluating the progress of tendon healing and improving the treatment of tendon injuries. However, radiologic quantitative assessment of tendon vascularity is not yet well established [5].

Contrast-enhanced ultrasound (CEUS) is a reliable method for observing microcirculation by ultrasound [6]. Sulfur hexafluoride microbubbles of the contrast agent are almost as large as erythrocytes and pass through the capillaries after injection [7]. Therefore, the tissue blood supply can be assessed by detecting the tissue microcirculation after the injection. Since 2012, musculoskeletal CEUS examinations have been applied to assess microperfusion of the tendon and muscle [8, 9]. Specifically, it was demonstrated that vascularity alterations in human Achilles tendinopathy and repaired rotator cuffs could be determined by CEUS [10, 11], and early retear after supraspinatus tendon repair and functional shoulder outcome could be predicted by CEUS [9, 12]. Though these studies correlate CEUS to the tendon blood supply and have opened new frontiers for CEUS applications, few of them relied on the histological and immunohistochemical assays as the gold standards to verify the efficacy of CEUS for assessing surgical repair outcomes. Little information has been focused on assessing the correlation of CEUS changes with tendon pathological and biomechanical changes during tendon healing. Furthermore, there are multiple quantitative parameters of CEUS, but few studies have evaluated which ones are truly effective in evaluating tendon healing.

Thus, the objective of this study was to perform an in vivo experiment on the rat patellar tendons to estimate if CEUS could be a suitable technique for quantitative measurement of angiogenesis during patellar tendon repair. The aims of our study were two-fold: the first was to select the suitable quantitative parameters for reflecting the time-dependent change in the blood supply of the healing site, and the second was to assess the correlation between the quantitative parameter and the histology as well as biomechanical properties of the healing tendon tissue.

## 2. Materials and Methods

### 2.1. Animal Experiments

The animal experiments were approved by the Animal Welfare and Ethics Group, Department of Laboratory Animal Science, Fudan University (no. 202206026Z). A total of 40 Sprague–Dawley rats (age 6 weeks, weight 190–210 g) were grouped randomly into control (*n* = 10) and operation (*n* = 10 at each time point) groups. All the rats were anesthetized through intraperitoneal injection of pentobarbitone sodium (30 mg/kg). In the operation group, both of the patellar tendons were exposed. Then a 7 mm × 2 mm full-thickness window defect was created in the central third of the patellar tendon without any bony defects. The wounds were washed with saline and closed with 6–0 silk sutures layer-by-layer [13–15]. After surgery, the rats were free to move in the cage without immobilization. The rats in the operation group (*n* = 10 at each time point) were anesthetized 7, 14, and 28 days after the operation for ultrasound examinations and sacrificed for histologic and biomechanical evaluations. The rats in the control group (*n* = 10) were anesthetized on day 0 for ultrasonic examinations and sacrificed for histologic and biomechanical examination as reference. All the right patellar tendons were employed for ultrasonography and histologic evaluations, and all the left patellar tendons were employed for the mechanical assessment.

### 2.2. Ultrasound Examination

At the predetermined time, the rats both in the operation group and control group (*n* = 10) were anesthetized, and the knees which had the body hair removed were fixed on a flat plate with tibias closing to the femur so that the patellar tendons were parallel to the flat plate. An acoustic coupler with a gel pad was placed between the knee and the probe to stabilize the operation.

Ultrasound examination was performed by a sonographer with 12 years of experience. First, the B-mode imaging was acquired by an Aplio i900 (Canon Medical Systems, Japan) with a 24-MHz linear transducer (i24LX8, Aplio i900, Canon Medical Systems, Japan). The machine parameters were adjusted in the grayscale mode (mechanical index: 0.9, gain: 63, dynamic range: 65, and frame rate: 27 fps). After scanning longitudinally and transversely, the largest cross-sectional area of the patellar tendon was selected, and the maximum thickness of the patellar tendon was measured.

The CEUS imaging was acquired by the same ultrasound instrument with an 18-MHz linear transducer (i18LX5, Aplio i900, Canon Medical Systems, Japan) after the B-mode scanning. The long axis of the probe was parallel with the long axis of the patellar tendon. The central section of the long axis of the patellar tendon was detected on the duplex ultrasound mode(grayscale parameters: mechanical index, 0.04; frequency, 6 MHz; frame rate, 10 fps; gain, 7; dynamic range, 60; contrast parameters: mechanical index, 0.08; harmonic frequency, 6 MHz; frame rate, 10 fps; gain, 60; and dynamic range, 60). After the technician injected the 0.2 ml SonoVue® (sulfur hexafluoride microbubble, Bracco, Italy) into the rat via the tail vein, videos were recorded immediately for 90 sec in the DICOM format [16]. The area of the patellar tendon was traced as the region of interest. Time-intensity curve (TIC) was automatically calculated by the software (Time Curve Analysis V3.7, Canon, Japan), yielding the following quantification parameters [17]: basic intensity (BI), defined as the basic signal intensity; peak intensity (PI), defined as the maximum signal intensity; area under the curve (AUC), defined as the area under TIC; time to peak (TTP), defined as the time it takes to reach PI from the beginning of enhancement; and Slope, defined as the slope of the midpoint position of the rising curve, representing the average rate of contrast agent perfusion.

### 2.3. Immunohistochemistry

At the predetermined time, the rats were sacrificed by overdose. The patellar tendons were harvested and immediately fixed in 4% paraformaldehyde. Samples were dehydrated, embedded in paraffin for sectioning at 4-*μ*m thick, and then stained with CD31. For immunohistochemistry, sections were stained using a primary antibody specific for CD31 (1 : 100, Abcam, UK), which is a marker of vascular endothelial cells [13]. Stained sections were viewed using an inverted optical light microscope (DM2500P, Leica, German), and digital images were captured with a microscopic imaging system (MicroPublisher 5.0RTV, QImaging, Canada) and its accompanying software, OLYSIM. The CD31-positive staining area and the microvessel diameter were calculated using Image J software.

### 2.4. Biomechanical Test

The bone-patellar tendon-bone complexes of the rats were harvested after sacrifice at the predetermined time. Specimens consisted of the lower femur, the patella tendon, and the tibia. The muscles and ligaments other than the patellar tendon ligament were removed. An electronic universal material testing machine (Instron5966, USA) was used for biomechanical testing. Each complex was fixed in the load frame with the fiber direction of the patellar tendon, which coincided with the stretching direction of the device. Subsequently, a tensile force was applied to the complex at an extension rate of 2 mm/min and recorded using a software package kit (Bluehill Universal, USA) [13]. The failure load (N) and tensile strength (MPa) were acquired from the load-deformation curve (*n* = 10 at each time point) when the patellar tendon ruptured.

### 2.5. Statistical Analysis

All statistical analyses were performed using IBM SPSS Statistics 22.0 (IBM, USA). Results were presented as the mean ± standard deviation (SD), with *p* < 0.05 considered statistically significant. Normal distribution of continuous variables in each group was assessed by the Kolmogorov–Smirnov (K-S) test. Their variance homogeneity was assessed by the Levene test. If *p* values of both K-S test and Levene test were greater than 0.05, one-way analysis of variance (ANOVA) would be conducted; otherwise, Kruskal–Wallis H test would be performed. Pearson correlation analysis was used for the correlation analysis of normally distributed data, whereas Spearman correlation analysis was used to test the associations between nonnormally distributed parameters.

## 3. Results

### 3.1. Ultrasound Imaging Analysis

**Figure 1:**
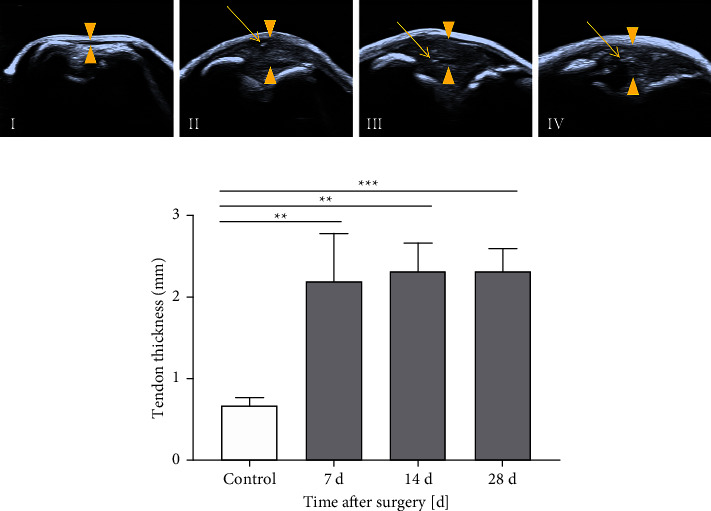
B-mode ultrasound imaging of the patellar tendons in the control and operation groups. (a) The structures of I control group, II 7 days, III 14 days, and IV 28 days after surgery are shown clearly. The triangles point out the maximum thickness of the patellar tendons. The arrows point to strong echoes from suture lines. (b) All of the tendons thickened after surgery. Data are expressed as the mean ± SD (*n* = 10 rats at each time point). ^*∗∗*^*p* < 0.01; ^*∗∗∗*^*p* < 0.001.

In B-mode, the structures of the patellar tendons were displayed, as shown in Figure 1(a). In the control group, the patellar tendons were hypoechoic and in sharp contrast to the surrounding hyperechoic soft tissues. In the operation group, the patellar tendons were heterogeneous echoic and surrounded by a thin hyperechoic border. Surgical sutures were displayed as punctate and short-linear strong echoes inside the tendons. All the patellar tendons of the operation group were significantly thickened compared with those of the control group (*p* < 0.01). There was no significant difference in the maximum thicknesses of the patellar tendon at each time point after surgery (*p* > 0.05), see Table 1 and Figure 1(b).

**Figure 2:**
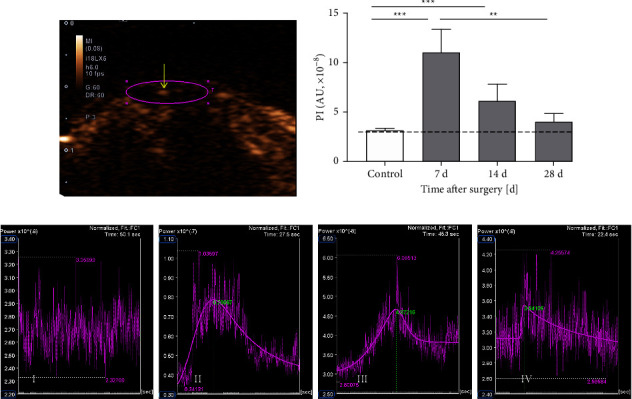
CEUS of the patellar tendons in the control and operation groups. (a) CEUS imaging of an exemplary injured patellar tendon 7 days after surgery. The circle delineates the patellar tendon as the ROI, and the arrow points to the spot-like enhancement in the center of the patellar tendon. (b) Statistical analysis of PI in CEUS. The dashed line indicates the BI. Data are expressed as mean ± SD of 10 rats at each time point. Please note that the data in the control group was the BI, not the PI. (c) The TIC of I control group, II 7 days, III 14 days, and IV 28 days after surgery. Please note that the patellar tendons of the control group were not enhanced. The high-frequency chaotic curve shown in the I control group was the basic intensity curve. The TIC of II 7 days, III 14 days, and IV 28 days after surgery could be fitted into smooth curves. ^*∗∗*^*p* < 0.01; ^*∗∗∗*^*p* < 0.001.

In the CEUS mode, the soft tissue around the patellar tendon was significantly enhanced after injection of the contrast agent, while microcirculation of the patellar tendons was reflected by different degrees of enhancement. In the control group, the patellar tendon consistently showed no enhancement. In the operation group, the patellar tendons were shown either internal scattered mild enhancement or branching moderate low enhancement, as shown in Figure 2(a) and Supplementary file 1. The quantitative analysis of CEUS based on TIC was displayed in Table 1, Figures 2(b) and 2(c). The TIC of the control group showed high-frequency chaotic curves, which was the basic intensity (BI) of the CEUS. The TIC of the operation group in the detection period could be fitted into bell-shaped smooth curves. Compared with the BI in the control group, the PI values increased in the operation group with a maximum at 7 days after the surgery (*p* < 0.001) and then gradually decreased. At 28 days after the surgery, the PI of the operation group was not significantly different from that of the control group (*p* > 0.05), which was equivalent to the BI. Other CEUS parameters (AUC, TTP, and Slope) had no group difference among 7, 14, and 28 days after the surgery (*p* > 0.05).

### 3.2. Immunohistochemical Analysis

**Figure 3:**
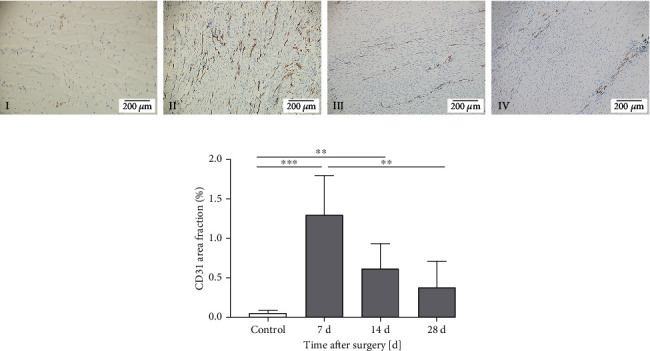
Immunohistochemistry of the patellar tendons in the control and operation groups. (a) Representative CD31 staining images at 100× magnification under a light microscope of I control tendon, II tendon 7 days, III 14 days, and IV 28 days after surgery. (b) The quantitative analysis of CD31-positive staining area fraction revealed highly angiogenic changes 7 days after surgery and decreased gradually. Data are expressed as mean ± SD of 10 rats at each time point. ^*∗∗*^*p* < 0.01; ^*∗∗∗*^*p* < 0.001.

The angiogenic property of the patellar tendons was evaluated ex vivo by immunohistochemistry with vascular endothelial cell marker CD31. In the control group, only few CD31 stained endothelial cells were observed. As shown in Figure 3(a), the CD31 stained endothelial cells could be observed at each time point in the operation group, and some CD31-positive microvessels could be visualized. As shown in Table 1 and Figure 3(b), the CD31 positive-staining area fraction increased in the operation group with a maximum at 7 days after the surgery (*p* < 0.001) and then gradually decreased. At 28 days after the surgery, the CD31 positive-staining area fraction of the operation group was not significantly different from that of the control group (*p* > 0.05). This angiogenic change was consistent with the change of the PI in CEUS. In addition, the quantitative analysis of the microvessel diameter showed no significant difference among 7, 14, and 28 days after the surgery (*p* > 0.05), as shown in Table 1.

### 3.3. Biomechanical Test Results

**Figure 4:**
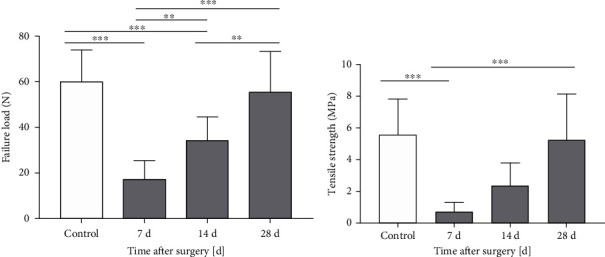
Mechanics indexes of the patellar tendons in the control and operation groups. (a) Failure loads and (b) tensile strength of patellar tendons after the surgery increased over time and had returned to near normal. Data are expressed as mean ± SD of 10 rats at each time point. ^*∗∗*^*p* < 0.01; ^*∗∗∗*^*p* < 0.001.

The biomechanical test assessed the quality of the repaired tendon. As shown in Table 1 and Figure 4, both the failure load and tensile strength of the repaired patellar tendons in the operation group presented the minimum at 7 days after the surgery (*p* < 0.001), and then continuously increased over time until approaching those of the control group. The failure load at 28 days after the surgery had no significant difference compared with that of the control group (*p* > 0.05), as shown in Figure 4(a). The tensile strength at 14 days after the surgery had no significant difference compared with that of the control group, and it was closer to the control group at 28 days after the surgery (*p* > 0.05), as shown in Figure 4(b).

### 3.4. Correlations

**Figure 5:**
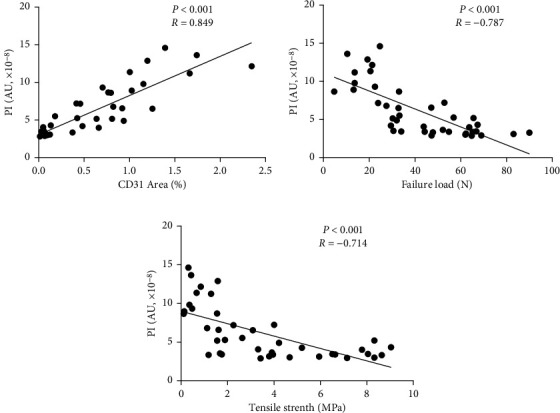
Statistical analysis correlating CEUS imaging, histology, and biomechanical properties. (a) Correlation analysis revealed a significant positive correlation between PI values and CD31-positive staining areas. (b) The PI value showed negative correlations with the failure load. (c) The PI value showed negative correlations with the tensile strength.

The relationship between the CEUS index and the progress of tendon healing was described through the correlation analysis. The PI was positively correlated with CD31-positive staining area fraction (*R* = 0.849, *p* < 0.001). In addition, the PI was negatively correlated with the failure load (*R* = −0.787, *p* < 0.001) and tensile strength (*R* = −0.714, *p* < 0.001), see Figures 5(a)–5(c).

## 4. Discussion

The most important finding of the present study was that the CEUS-derived parameter, PI, could quantify angiogenic changes during tendon healing and reflect functional recovery to a certain extent.

B-mode ultrasound could noninvasively show the delicate structures of tendons, which provided a good foundation for locating the injured areas and delineating a region of interest (ROI) for CEUS. In the operation group, all the patellar tendons were significantly thickened at each time point due to the edema [18], so the internal structure could be observed more clearly. The internal echoes of the tendons in the operation group were uneven, which could be related to the inflammation response after the surgery [19]. As given in Figure 1(a), the surgical suture echoes could be used as signs to identify the injured areas. The tendon boundary was a sign to delineate the ROI for CEUS. In the operation group, the thin hyperechoic capsule around the tendon could be used to distinguish the tendon boundary. In the control group, even if the patellar tendons were too thin to display the capsules, their boundary could be identified by the contrast to the surrounding soft tissues.

CEUS could be used to show the blood flow signals inside the injured tendon of rats in vivo, as it can detect blood flow signals from microvessels as small as 7–10 *μ*m [6]. In this study, the mean diameter of the vessels in the operation group was 7–7.14 *μ*m and that of the contrast agent microbubble was 2.5 *μ*m [20]). Therefore, although the tendon has a limited blood supply [21], the microcirculation of the injured tendon could still be displayed. The mean ultrasound signal intensity induced by contrast uptake was linearly linked to the number of microbubbles in the ROI [22]. Thus, the degree of tendon enhancement on CEUS was low or mild may be due to the small number and thin diameter of the microvessels, which limited the number of ultrasound contrast agent microbubbles passing through. And the microvessel diameters were similar after the surgery in this study (*p* > 0.05), which concluded that the difference in the PI at each time point may be due to the different number of microvessels, rather than the diameter. In the control group, the tendons showed no enhancement due to the absence of angiogenesis.

CEUS can provide real-time, continuous dynamic imaging, and TIC can provide a quantitative analysis of local contrast enhancement [23]. As displayed in Figures 2(c) and 3(a), the basic intensity curves displayed in the control group reflected the absence of blood perfusion in tendons, which was confirmed by the absence of microvessels in immunohistochemistry. The bell-shaped curves fitted in the operation group revealed the recovery of wash-in and wash-out pattern of blood perfusion [13], which was confirmed by the presence of the microvessels in immunohistochemistry.

According to the quantitative analysis of the CEUS parameters, only the PI reflected the time-dependent changes in the tendon blood supply in this study, as given in Table 1. The PI value represents the transient maximum concentration of contrast agent microbubbles in the tissue, which is proportional to the average blood volume in the region of interest and reflects the blood flow of the local tissue [8]. In this study, the change of the PI was consistent with the change of CD31-positive area fraction of the rat patellar tendon, and they were significantly correlated (*R* = 0.849, *p* < 0.001), as given in Figures 2(b), 3(b), and 5(a). CD31 is one of the characteristic makers of newly formed vascular [24]. The highly expressed CD31 indicates active proliferation of endothelial cells [25]. As displayed in Figure 3(b), the change of CD31-positive area fraction showed highly angiogenic changes at 7 days after the surgery and then decreased, and this change was in line with the change of *α*-SMA-positive microvessel density (MVD) and VEGF-positive area fraction of injured rat patellar tendons in a previous study [5]. *α*-SMA and VEGF are, like CD31, widely used to assess angiogenesis [25–27]. Therefore, it confirmed that the change of the PI value could synchronously reflect the angiogenesis change during patellar tendon healing.

The biomechanical test was used to verify functional recovery of the patellar tendon. Previous studies demonstrated that vascularization during tendon healing is directly related to the biomechanical properties of the tendon [28, 29]. However, a suitable technique for a noninvasive in vivo monitoring of the biologic healing processes has not been established yet [5, 30]. In this study, the PI showed negative correlations with the failure load and tensile strength respectively, which showed that the PI value could reflect functional recovery of the patellar tendon to a certain extent. Hence, quantitative analysis of CEUS is a possible solution for evaluating the progress of tendon healing and has a clinical value.

It should be noted that this study had some limitations. Firstly, the whole perfused tendon image was unavailable since the ultrasound beam could only scan one section simultaneously. Thus, always the central section of the long axis of the patellar tendon that reflected the blood microcirculation near the injury site to the greatest extent was measured in this study. Secondly, the rupture of the patellar tendon in the rat model was different from that occurs in the more complex clinical condition. The current study just demonstrated the technical feasibility of quantitative analysis of CEUS. Further experiments are necessary to improve its clinical application. Thirdly, during the healing process of the tendon, a variety of regulatory growth factors are called up to promote angiogenesis [31]. However, only one angiogenic biomarker (CD31) was used in this study. The correlation between CEUS intensity and other more angiogenic markers would be studied in the future to obtain a more comprehensive evaluation.

## 5. Conclusion

This study showed that the CEUS-derived parameter, PI, could quantitatively reflect the time-dependent change in the blood supply of the healing site, and the PI correlated with histologic and biomechanical properties of the healing tendon. Thus, quantitative analysis of CEUS could be a promising means applied in quantifying angiogenesis in tendon healing and might be a new efficient tool in vivo monitoring tendon healing in experimental studies and for potential clinical use in the future.

## Figures and Tables

**Table 1:** Summary of ultrasonic, histologic, and biomechanical assessment in the control, day 7, 14, and 28 (Mean ± SD).

Measurements	Control	Day 7	Day 14	Day 28	*p* value
Maximum thickness (mm)	0.67 ± 0.10	2.20 ± 0.59	2.31 ± 0.35	2.31 ± 0.28	<0.001
PI (AU, ×10^−8^)	3.16 ± 0.22^*∗*^	11.19 ± 2.14	6.18 ± 1.68	4.15 ± 0.74	<0.001
AUC (AU.s, ×10^−8^)	—	440.10 ± 359.06	270.90 ± 259.28	132.90 ± 142.72	0.053
TTP (s)	—	4.15 ± 0.74	4.91 ± 2.54	9.22 ± 7.37	0.132
Slope (AU/s, ×10^−8^)	—	17.91 ± 48.16	4.19 ± 4.08	1.57 ± 1.79	0.061
Microvessel diameter (*μ*m)	—	6.97 ± 3.13	6.68 ± 4.07	7.05 ± 2.47	0.068
CD31 area (%)	0.06 ± 0.03	1.31 ± 0.49	0.62 ± 0.31	0.38 ± 0.33	<0.001
Failure load (N)	60.15 ± 13.77	17.38 ± 8.02	34.33 ± 10.25	55.58 ± 17.70	<0.001
Tensile strength (MPa)	5.57 ± 2.27	0.72 ± 0.57	2.37 ± 1.42	5.27 ± 2.87	<0.001

^*∗*^This value was the mean (±SD) of the highest values of the BI.

## Data Availability

The data used to support the findings of this study are available from the corresponding authors upon request.

## Supplementary Materials

Supplementary Materials Supplementary file 1: https://youtu.be/vgyCalv3Xmo.
