# Supplementary material for: Quantitative Analysis of Contrast-Enhanced Ultrasound That Can Be Used to Evaluate Angiogenesis during Patellar Tendon Healing in Rats
Source: Contrast Media Mol Imaging. 2022 Oct 13;2022:6867743. doi: 10.1155/2022/6867743 (PMC9584743; doi:10.1155/2022/6867743)
Supplement: Supplementary Materials — Supplementary file 1: https://youtu.be/vgyCalv3Xmo. [file 6867743.f1.docx]

**Supplementary materials**

Supplementary file 1: https://youtu.be/vgyCalv3Xmo

The purple circle presented the region of interest. The patellar tendon area was first outlined by the surrounding enhancing soft tissue as a near-elliptical hypoechoic zone. Then the contrast microbubbles moved from the outside to the inside of the tendon in a punctate and branching pattern, making the tendon locally low to moderate enhanced.
